# Clinical significance of VEGF-C status in tumour cells and stromal macrophages in non-small cell lung cancer patients

**DOI:** 10.1038/sj.bjc.6601992

**Published:** 2004-06-29

**Authors:** E Ogawa, K Takenaka, K Yanagihara, M Kurozumi, T Manabe, H Wada, F Tanaka

**Affiliations:** 1 Department of Thoracic Surgery, Faculty of Medicine, Kyoto University, Kyoto, Japan; 2 Department of Translational Clinical Oncology, Kyoto University, Kyoto, Japan; 3 Laboratory of Anatomic Pathology, Kyoto University, Kyoto, Japan

**Keywords:** angiogenesis, lymphangiogenesis, stromal macrophages, VEGF, prognosis

## Abstract

Recent experimental studies have revealed that tumour-associated stromal macrophages as well as tumour cells express vascular endothelial growth factor C (VEGF-C), which plays important roles in lymphangiogenesis, which is a critical factor in the progression of many malignant tumours including non-small-cell lung cancer (NSCLC). However, no clinical study on VEGF-C expression in both stromal macrophages and tumour cells has been reported, and we conducted the present study to address the issue in resected NSCLC. A total of 206 patients with completely resected pathologic stage I–IIIA NSCLC were retrospectively reviewed. Expression of VEGF-C in primary lung tumour was assessed immunohistochemically. Expression of VEGF-C in tumour cells was high in 125 patients (60.7%), and that in stromal macrophages was positive in 136 patients (71.2%). The status of VEGF-C in tumour cells or in stromal macrophages was not correlated with nodal status or angiogenesis. The 5-year survival rate of high tumoral VEGF-C patients (60.7%) was significantly lower than that of low tumoral VEGF-C patients (39.3%) (*P*=0.046), and a multivariate analysis confirmed that tumoral VEGF-C status was a significant and independent prognostic factor. Moreover, tumour showing high VEGF-A and VEGF-C expression in tumour cells showed the poorest prognosis (5-year survival rate, 45.1%). The status of VEGF-C in stromal macrophages was not correlated with the prognosis. In conclusion, tumoral VEGF-C status, not stromal VEGF-C status, was a significant prognostic factor in resected NSCLC.

Primary lung cancer is the leading cause of cancer deaths in most industrialised countries, and non-small-cell lung cancer (NSCLC) accounts for 75–80% of primary lung cancer. Tumour node metastasis (TNM) factors are generally used in the evaluation of tumour progression, and nodal involvement (N-factor) as well as distant metastasis (M-factor) is the critical factor to determine the prognosis of NSCLC (Mountain, 1997; Naruke *et al*, 1998; Tanaka *et al*, 2000). In addition, some clinical studies demonstrated that lymphatic invasion is also a prognostic factor in NSCLC (Macchiarini *et al*, 1993; Brechot *et al*, 1996). Thus, lymphatic spread is a critical factor to determine the progression and prognosis.

The vascular endothelial growth factor (VEGF) family is a group of growth factors that regulate the growth of endothelial cells (ECs). Among VEGF family members, it has been well known that VEGF-A is the most potent angiogenic factor and plays important roles in the progression of malignant tumours (Folkman, 1995). Recently, VEGF-C has been identified as a new VEGF family member (Paavonen *et al*, 1996; Cao *et al*, 1998; Yonekura *et al*, 1999), and it has been experimentally revealed that VEGF-C mediates lymphangiogenesis (Makinen *et al*, 2001; Mandrisota *et al*, 2001; Skobe *et al*, 2001). More recently, Padera *et al* (2002) have revealed in an experimental mouse model that VEGF-C expression correlates with the incidence of lymph node metastases. These results strongly suggest that VEGF-C can be an important diagnostic and therapeutic target for treating malignant tumours (Pepper, 2001), but clinical significance of VEGF-C expression has been established.

In NSCLC, only a few clinical studies on VEGF-C expression have been reported (Niki *et al*, 2000; Ohta *et al*, 2000; Kajita *et al*, 2001; Arinaga *et al*, 2003), and the prognostic significance of VEGF-C status remains controversial. In addition, in all these studies, VEGF-C expression was analysed only in tumour cells, whereas recent experimental and clinical studies revealed that stromal cells, especially stromal macrophages, did express VEGF-C and played important roles in peritumoral lymph angiogenesis (Schoppmann *et al*, 2002; Krishnan *et al*, 2003). Thus, in the present study, we assessed for the first time VEGF-C expression in stromal macrophages as well as in tumour cells in correlation with clinical outcomes in resected NSCLC.

## PATIENTS AND METHODS

### Patients and tissue preparation

A total of 206 patients with pathologic (p-) stage I–IIIA NSCLC who underwent complete tumour resection without any preoperative therapy at Kyoto University Hospital between January 1985 and December 1990 and whose histological specimens are available for immunohistochemical staining (IHS) were retrospectively reviewed (Table 1

**Table 1:** Expression of VEGF-C in stromal macrophages and tumour cells in resected NSCLC

	**Expression of VEGF-C in**					
	**Stromal macrophages**			**Tumour cells**		
	**Negative**	**Positive**	***P*-value**	**Low**	**High**	***P*-value**
All patients	55 (28.8)	136 (71.2)		81 (39.3)	125 (60.7)	
						
Age (mean)	63.8 years	62.3 years	0.324	62.7 years	62.5 years	0.884
Lower (<64 years)	26 (28.3)	66 (71.7)	1.000	39 (38.6)	62 (61.4)	0.887
Higher (⩾64 years)	29 (29.3)	70 (70.7)		42 (40.0)	63 (60.0)	
						
*Gender*						
Male	44 (31.4)	96 (68.6)	0.124	59 (39.1)	92 (60.9)	1.000
Female	11 (21.6)	40 (78.4)		22 (40.0)	33 (60.0)	
						
*Performance status*						
0	48 (28.9)	118 (71.1)		71 (39.9)	107 (60.1)	
1	7 (30.4)	16 (69.6)	0.657	10 (38.5)	16 (61.5)	0.515
2	0 (0.0)	2 (100)		0 (0.0)	2 (100)	
						
*Histologic type*						
Squamous cell	21 (30.4)	48 (69.6)	0.733^a^	31 (40.8)	45 (59.2)	0.761^a^
Adenocarcinoma	29 (27.6)	76 (72.4)		42 (37.8)	69 (62.2)	
Large cell	2 (18.2)	9 (81.8)		5 (45.5)	6 (54.5)	
Others	3 (50.0)	3 (50.0)		5 (62.5)	3 (37.5)	
						
*Tumour differentiation*						
Poor	11 (25.6)	32 (74.4)		19 (39.6)	29 (60.4)	
Moderate	24 (30.0)	56 (70.0)	0.864	33 (39.8)	50 (60.2)	0.992
Well	17 (27.4)	45 (72.6)		26 (38.8)	41 (61.2)	
						
*Pathologic stage*						
I	35 (32.4)	73 (67.6)		48 (40.7)	70 (59.3)	
II	6 (30.0)	14 (70.0)	0.363	12 (54.5)	10 (45.5)	0.151
IIIA	14 (22.2)	49 (77.8)		21 (31.8)	45 (68.2)	
						
*Pathologic (p-) T-factor*						
pT1	24 (35.8)	43 (64.2)		33 (45.8)	39 (54.2)	
pT2	25 (25.5)	73 (74.5)	0.280	41 (38.7)	65 (61.3)	0.157
pT3	6 (23.1)	20 (76.9)		7 (25.0)	21 (75.0)	
						
*Pathologic (p-) N-factor*						
pN0	38 (30.9)	85 (69.1)		52 (38.5)	83 (61.5)	
pN1	7 (31.8)	15 (68.2)	0.477	13 (54.2)	11 (45.8)	0.338
pN2	10 (21.7)	36 (78.3)		16 (34.0)	31 (66.0)	

Each figure shows the number of patients, and the percentage is shown in parentheses.

a Comparison between squamous cell carcinoma and adenocarcinoma.

). Pathologic stage was re-evaluated and determined with the present TNM classification as revised in 1997 (Mountain, 1997). Histological type and cell differentiation were determined using the current classification by WHO as revised in 1999 (Travis *et al*, 1999). For analyses according to the differentiation of cancer cells, well-differentiated squamous cell carcinoma (Sq) and adenocarcinoma (Ad) were classified as well-differentiated tumours and moderately differentiated Sq and Ad as moderately differentiated tumours; large cell carcinoma (La) and poorly differentiated Sq and Ad were classified as poorly differentiated tumours, and the other histologic types were excluded in the analyses. For all of these patients, records of surgery, the in-patient medical records, chest X-ray films, whole-body computed tomography (CT) films, and bone scanning films were reviewed. Follow-up of postoperative clinical course was conducted by outpatient medical records and by inquiries by telephone or letter.

All of the primary tumour specimens were immediately fixed in 10% (v v^−1^) formalin, and then embedded in paraffin. Serial 4-*μ*m sections were prepared from each sample, and served for haematoxylin and eosin (HE) staining, the terminal deoxynucleotidyl transferase (TdT)-mediated dUTP–biotin nick end-labelling (TUNEL) staining, and IHS. Slides were reviewed independently by two investigators (EO and KT) without a knowledge of any clinical data. This study was approved by the Institutional Review Board of Kyoto University.

### Immunohistochemical staining

Expression of VEGF-C was evaluated with IHS using a streptavidin–biotinylated horseradish peroxidase detection system (LSAB+kit/HRP; DAKO, Kyoto, Japan). After retrieval of the antigen with heating in a microwave oven for 15 min, sections were incubated overnight at 4°C with an anti-VEGF-C goat polyclonal antibody (N-19; Santa Cruz, San Diego, CA, USA) diluted at 1 : 50. For the negative control, the primary antibody was omitted. As a chromogen, diaminobenzidine-tetrahydrochloride (0.03%) containing 0.1% hydrogen peroxide was used, and sections were counterstained with haematoxylin. The expression of VEGF-C in tumour cell was classified based on the staining intensity as follows: score 0 if no staining was detected; score 1 if the staining intensity was weak; score 2 if the intensity was moderate; score 3 if the intensity was high; VEGF-C status in tumour cells was finally classified as low expression (score 0 or 1) or high expression (score 2 or 3). The expression of VEGF-C in stromal macrophages was classified as negative or positive staining. For exact identification of stromal macrophages, serial sections were used for IHS for VEGF-C expression and IHS for CD68, which had been reported to be a specific marker of macrophages; a mouse anti-CD68 monoclonal antibody (clone KP1; DAKO, Kyoto, Japan) diluted at 1 : 2000 was used. Specificity of VEGF-C expression and identification of stromal macrophages were confirmed by two pathologists (KM and MT).

Expression of VEGF-A was also evaluated immunohistochemically as described previously (Tanaka *et al*, 2001). Briefly, an anti-VEGF-A goat polyclonal antibody (A-20; Santa Cruz) diluted at 1 : 50 was used as the primary antibody, and status of VEGF-A expression was classified based on the staining intensity and the percentage of positive-staining tumour cells as low expression or high expression (Tanaka *et al*, 2001).

Intratumoral microvessel density (IMVD), a measurement of tumour angiogenesis, was evaluated immunohistochemically using a mouse monoclonal antibody (QBEnd10, diluted at 1 : 50; DAKO) against CD34, a pan-endothelial marker and a mouse monoclonal antibody (SN6h, diluted at 1 : 100; DAKO) against CD105, a specific marker of activated ECs, as described previously (Tanaka *et al*, 2001). The 10 most vascular areas within a section were selected for evaluation of angiogenesis, and vessels labelled with the anti-CD34 antibody or the anti-CD105 antibody were counted under light microscopy with a 200-fold magnification. The average counts were recorded as the CD34-IMVD or the CD105-IMVD for each case.

Proliferative activity of tumour cells and p53 status were also evaluated with IHS as described previously (Tanaka *et al*, 1999); an anti-proliferative cell nuclear antigen (PCNA) monoclonal antibody PC-10 (mouse IgG2a, kappa, 400 *μ*mg ml^−1^; DAKO) diluted at 1 : 50 and an anti-p53 monoclonal antibody DO-7 (mouse IgG2b, kappa, 250 *μ*g ml^−1^; DAKO) diluted at 1 : 50 were used as primary antibodies, respectively. A total of 1000 tumour cells were counted, and the percentages of positive cells were determined. Proliferative index (PI) was defined as the percentage of PCNA-positive cells (%); when the percentage of positive-staining cells exceeded 5%, the slide was judged to exhibit aberrant expression of p53.

### TUNEL staining

Detection of apoptotic cells was performed with the TUNEL method as described previously (Tanaka *et al*, 1999). The TUNEL staining was performed using the *In Situ* Death Detection Kit, POD (Boehringer Manheim, Manheim, Germany) following the manufacturer's protocol. The specificity of the TUNEL staining of apoptotic cells was confirmed by making the negative and the positive control slides at every staining. As negative control slides, sections incubated with the TUNEL reaction mixture without TdT were used. As positive control slides, sections treated with 0.7 mg ml^−1^ DNase I (Stratagene, La Jolla, CA, USA) for 10 min at 25°C before the TUNEL reaction were used. Apoptotic cells were determined with careful observation of TUNEL-staining sections and serial HE-staining sections, and TUNEL-staining cells, if they represented the histological features of necrosis in HE-staining sections, were not considered to be apoptotic cells. In each case, a total of 10 000 tumour cells, consisting of 1000 tumour cells each in 10 different fields, were evaluated at high magnification (× 400).

### Statistical methods

Counts were compared by the *χ*2 test. Continuous data were compared using Student's *t*-test if the sample distribution was normal, or using Mann–Whitney *U*-test if the sample distribution was asymmetrical. The postoperative survival rate was analysed by the Kaplan–Meier method, and the differences in survival rates were assessed by the log-rank test. Multivariate analysis of prognostic factors was performed using Cox’s regression model. Differences were considered significant when *P* was less than 0.05. All statistical manipulations were performed using the SPSS for Windows software system (SPSS Inc., Chicago, IL, USA).

## RESULTS

### Expression of VEGF-C in NSCLC

**Figure 1:**
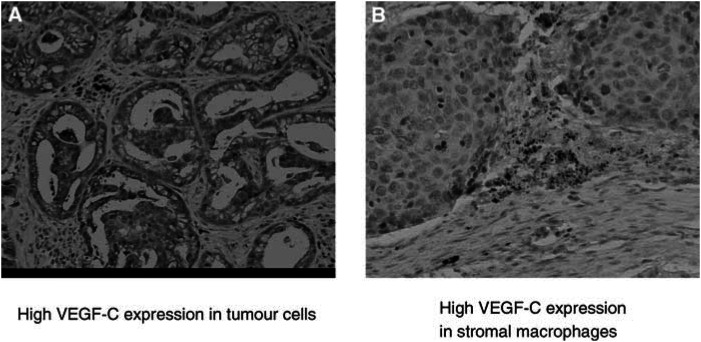
Expression of VEGF-C in NSCLC. Vascular endothelial growth factor C was observed in (**A**) tumour cells and (**B**) stromal macrophages.

Expression of VEGF-C was seen in the cytoplasm of tumour cells (Figure 1A

), and high VEGF-C expression in tumour cells was seen in 125 patients (60.7%) (Table 1). The status of VEGF-C in tumour cells was not correlated with any patients’ characteristics including nodal metastasis (Table 1).

The expression of VEGF-C was also seen in stromal macrophages (Figure 1B), and the expression was positive in 136 patients (71.2%) (Table 1). No significant correlation between any patients’ characteristic and VEGF-C status in stromal macrophages was documented (Table 1). A strongly positive correlation between tumoral VEGF-C status and stromal VEGF-C status was documented (*P*<0.001) (Table 2

**Table 2:** Biomarkers according to expression of VEGF-C in stromal fibroblasts and tumour cells in resected NSCLC

	**Expression of VEGF-C in**					
	**Stromal macrophages**			**Tumour cells**		
	**Negative**	**Positive**	***P*-value**	**Low**	**High**	***P*-value**
*VEGF-A expression in tumour cells*						
Low	35 (29.2)	85 (70.8)	1.000	59 (45.4)	71 (54.6)	0.026
High	20 (28.2)	51 (71.8)		22 (28.9)	54 (71.1)	
						
*VEGF-C expression in stromal macrophages*						
Low				36 (65.5)	19 (34.5)	<0.001
High				36 (26.5)	100 (73.5)	
						
*VEGF-C expression in tumour cells*						
Negative	36 (50.0)	36 (50.0)	<0.001			
Positive	19 (16.0)	100 (84.0)				
						
*Intratumoral microvessel density (IMVD)*						
CD34-IMVD (mean)	191.1	183.3	0.623	175.3	189.9	0.306
CD105-IMVD (mean)	41.4	45.1	0.643	38.4	47.7	0.190
						
*p53 aberrant expression*						
Negative	34 (31.2)	75 (68.8)	0.424	53 (44.9)	65 (55.1)	0.062
Positive	21 (25.6)	61 (74.4)		28 (31.8)	60 (68.2)	
						
Apoptotic index (mean)	14.5	19.3	0.130	17.9	17.8	0.977
Proliferative index (mean)	36.6	48.3	0.008	43.2	47.6	0.281

Each figure shows the number of patients, and the percentage is shown in parentheses.

).

### The status of VEGF-C and other biomarkers

A significantly positive correlation between VEGF-C status in tumour cells and VEGF-A status in tumour cells was documented (*P*=0.026). Tumours showing aberrant expression of p53 seemed to have higher incidence of high VEGF-C expression in tumour cells, but the difference did not reach a statistical significance (*P*=0.062) (Table 2). No significant correlation between VEGF-C status in angiogenesis, incidence of apoptosis, or proliferative activity was seen (Table 2).

The mean PI for tumour showing positive VEGF-C expression in stromal macrophages was significantly higher than that for tumour showing no VEGF-C expression in stromal macrophages. The status of VEGF-C in stromal macrophages was not correlated with any other biomarker including IMVD (Table 2).

### Postoperative survival

The 5-year survival rate of high tumoral VEGF-C patients (67.9%) was significantly lower than that of low tumoral VEGF-C patients (53.5%) (*P*=0.046; Table 3

**Table 3:** Postoperative survival according to expression of VEGF-C in NSCLC

	**Expression of VEGF-C in**					
	**Stromal macrophages**			**Tumour cells**		
	**Negative (%)**	**Positive (%)**	***P*-value**	**Low (%)**	**High (%)**	***P*-value**
All patients	57.3	60.3	0.921	67.9	53.5	0.046
						
*Stratified with pathologic (p-) stage*						
p-Stage I	61.1	74.3	0.246	76.0	65.5	0.231
p-Stage II	44.4	69.2	0.329	66.7	44.4	0.559
p-Stage IIIA	53.9	33.2	0.117	48.8	33.5	0.176
						
*Stratified with histologic type*						
Squamous cell	49.9	63.4	0.656	70.7	48.3	0.056
Adenocarcinoma	58.5	56.1	0.613	64.2	53.7	0.291

Each figure shows the 5-year survival rate of the each patient group.

and Figure 2

**Figure 2:**
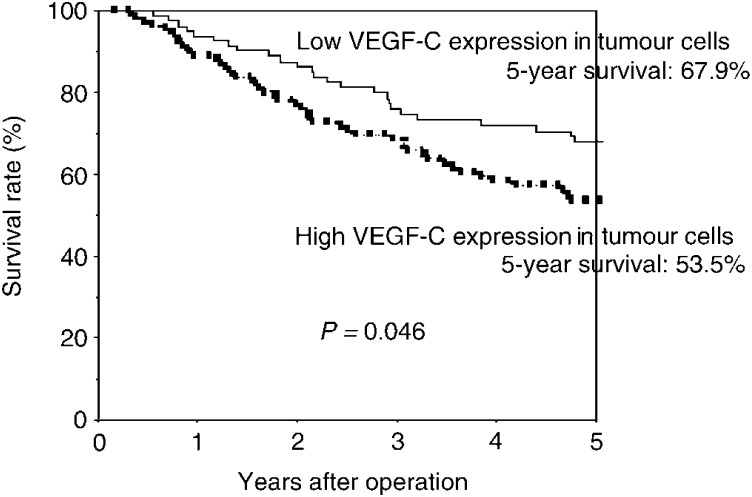
Postoperative survival of completely resected p-stage I–IIIA NSCLC. Comparison according to the status of VEGF-C expression in tumour cells: patients who had high staining for VEGF-C showed significantly less favourable survival rates compared with patients who had low staining for VEGF-C (*P*=0.046).

). There was no difference in the survival according to the VEGF-C status in stromal macrophages (Table 3). A multivariate analysis confirmed that high VEGF-C expression in tumour cells was a significant and independent prognostic factor in resected NSCLC (Table 4

**Table 4:** Multivariate analysis of prognostic factors in NSCLC

**Prognostic factors**	***β***	***P*-value**	**Relative hazard (95% confidence interval)**
Age	0.016	0.216	1.017 (0.991–1.043)
Sex (male/female)	−0.245	0.391	0.783 (0.447–1.370)
Performance status (0/1/2)	0.336	0.250	1.400 (0.790–2.476)
Histologic type (non-adenocarcinoma/adenocarcinoma)	−0.034	0.290	0.967 (0.909–1.029)
Pathologic stage (I, II, IIIa)	0.629	<0.001	1.876 (1.472–2.290)
High VEGF-C expression in tumour cells	0.545	0.021	1.724 (1.087–2.734)

).

**Figure 3:**
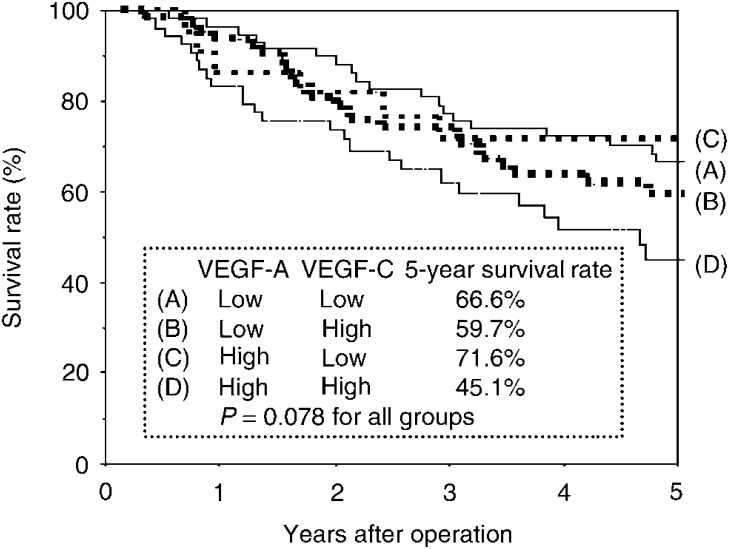
Postoperative survival of completely resected p-stage I–IIIA NSCLC. Comparison according to the status of VEGF-A expression and VEGF-C expression in tumour cells. The survival rates of patients who had high VEGF-A and VEGF-C expression in tumour cells and patients who had low VEGF-A and VEGF-C expression in tumour cell were 45.1 and 66.6%, respectively. The survival rates of patients who had high VEGF-A and low VEGF-C expression in tumour cells and patients who had low VEGF-A and high VEGF-C expression in tumour cell were 71.6 and 59.7%, respectively.

When combined with VEGF-A status in tumour cells, the prognostic impact of VEGF-C status in tumour cells was enhanced (Figure 3

). Tumour showing high VEGF-A and VEGF-C expression in tumour cells showed the poorest prognosis (5-year survival rate, 45.1%).

## DISCUSSION

We reported for the first time VEGF-C expression in stromal macrophages as well as in tumour cells in correlation with clinical outcomes in resected NSCLC. In previous clinical studies, it has been reported that VEGF-C status in tumour cells was significantly correlated with nodal metastasis and/or lymphatic vessel invasion in a variety of malignant tumours such as head and neck carcinoma (Oc *et al*, 2001), thyroid carcinoma (Bunone *et al*, 1999; Fellmer *et al*, 1999), oesophageal carcinoma (Kitadai *et al*, 2001; Noguchi *et al*, 2002), breast carcinoma (Kinoshita *et al*, 2001), gastric carcinoma (Yonemura *et al*, 1999, 2001; Kawashima *et al*, 2001), uterine carcinoma (Hashimoto *et al*, 2001; Hirai *et al*, 2001; Schoppmann *et al*, 2002), prostate carcinoma (Tsurusaki *et al*, 1999), and NSCLC (Niki *et al*, 2000; Ohta *et al*, 2000; Kajita *et al*, 2001; Arinaga *et al*, 2003). However, only one clinical study on VEGF-C expression in stromal macrophages has been reported, which showed that tumour-associated macrophages express VEGF-C and play important roles in peritumoral lymphangiogenesis in cervical cancer (Schoppmann *et al*, 2002). We also demonstrated in the present study that stromal macrophages did express VEGF-C in NSCLC. We failed to demonstrate any correlation between VEGF-C status in tumour cells or stromal cells and angiogenesis or nodal metastasis. To assess more accurately a correlation between VEGF-C status and nodal status, quantitative analysis of VEGF-C expression such as real-time reverse transcriptase–polymerase chain reaction for large-scale patient population should be conducted prospectively in future studies.

Some clinical studies revealed that VEGF-C status in tumour cells was a significant prognostic predictor in gastric carcinoma (Yonemura *et al*, 1999; Ichikura *et al*, 2001) and cervical carcinoma (Hirai *et al*, 2001), and the clinical impact remains unclear. In NSCLC, only a few studies have assessed the prognostic significance (Niki *et al*, 2000; Ohta *et al*, 2000; Kajita *et al*, 2001). Tumoral VEGF-C status was a significant prognostic factor in a univariate analysis, but a multivariate analysis failed to show a statistical significance in two studies (Niki *et al*, 2000; Kajita *et al*, 2001); a univariate analysis failed to show that tumoral VEGF-C expression was a significant prognostic factor in one study (Ohta *et al*, 2000). The present study showed that high VEGF-C expression in tumour cells was a significant and independent factor to predict a poor prognosis, and that tumour with high VEGF-A and VEGF-C expression in tumour cells showed the poorest prognosis. Unfortunately, we failed to show any clinical impact of VEGF-C status in stromal macrophages. To further assess clinical impact of VEGF-C status in tumour cells and stromal macrophages, expression status of the receptors such as VEGFR-3 should also be examined.

In conclusion, VEGF-C is expressed by both tumour cells and stromal cells in NSCLC, and VEGF-C status in tumour cells was significantly correlated with the prognosis. These findings suggest that VEGF-C may be an important target for diagnosis and/or therapy of NSCLC. Further investigations are necessary to clarify and understand the role of VEGF-C in patients with NSCLC.
